# Changes in moral decision-making during the COVID-19 pandemic

**DOI:** 10.1192/j.eurpsy.2021.1753

**Published:** 2021-08-13

**Authors:** S. Enikolopov, T. Medvedeva, O. Boyko, O. Vorontsova, O. Kazmina

**Affiliations:** Clinical Psychology, Federal State Budgetary Scientific institution “Mental health research center”, Moscow, Russian Federation

**Keywords:** Moral decision-making, COVID-19, Moral dilemmas, psychopathology

## Abstract

**Introduction:**

Stress can influence moral decisions.

**Objectives:**

The aim of the study was to evaluate whether the stress experienced by people during the COVID-19 pandemic can change moral decision making.

**Methods:**

311 respondents took part in the Internet survey 30.03.20-31.05.20, including SCL-90-R, and a subset of moral dilemmas proposed by Greene J.D (30 dilemmas in Russian), with «footbridge dilemma» among them as a personal dilemma and «trolley dilemma» as impersonal. The relationship of utilitarian personal dilemmas choices with psychopathological characteristics was analyzed. Personal moral dilemma choices were considered separately, in subgroups with a high level of somatization (N=107) and a high level of psychopathological symptoms (N=76).

**Results:**

The results showed an increase in personal dilemmas choices: 2.84 mean utilitarian choice in March - April and 3.17 in May (Univariate Analysis of Variance, age, gender as Covariates, p<0.01). At the beginning of the study the groups did not differ in the number of utilitarian personal choices, and at the end of the study the number of personal choices increased in the subgroup with a high level of psychopathology (4.7 utilitarian choices in May) and became statistically higher than in other groups (ANOVA with Bonferonni correction). In the subgroup with a high level of somatization, personal choices slightly decreased by the end of the survey (2.68 choices).

**Conclusions:**

The level of stress during the COVID-19 ambiguously affects moral decisions: a higher level of psychopathological symptoms leads to an increase in utilitarian choices and a high level of somatization leads to a decrease in utilitarian choices.

**Disclosure:**

No significant relationships.

